# Editorial: Cognitive Event-Related Potentials in Psychopathology: New Experimental and Clinical Perspectives

**DOI:** 10.3389/fpsyg.2016.01738

**Published:** 2016-11-07

**Authors:** Salvatore Campanella, Pierre Maurage

**Affiliations:** ^1^Laboratoire de Psychologie Médicale et d'Addictologie, Université Libre de BruxellesBruxelles, Beligium; ^2^Laboratoire de Psychopathologie Expérimentale, Institut de Recherche en Sciences Psychologiques, Université Catholique de LouvainLouvain-La-Neuve, Belgium

**Keywords:** event-related potentials, biomarkers, cognitive processes, psychopathology, relapse

A common feature of many psychopathological states (going from anxiety, depression to schizophrenia, or addictive states) is to be associated with large-scale cognitive impairments, which have a clear impact on the onset and maintenance of clinical symptoms (Menon, 2011). Therefore, studies have shown that the training and rehabilitation of cognitive skills lead to positive effects on patients' quality of life, centrally by decreasing the severity of these clinical symptoms (e.g., Pilling et al., 2002). However, beyond patent cognitive impairments, some minor cognitive restrictions can also be present and, even if not observable at the behavioral level, may induce a state of “vulnerability” that can, in some circumstances, facilitate the persistence of the psychopathology (Levit Binnun and Golland, 2012). In alcohol-related disorders for example, it is well-known that, despite a well-structured detoxification treatment encompassing psychiatric, psychological and pharmacological therapies, 50–90% of patients will relapse or restart consuming alcohol in the year following detoxification (Boothby and Doering, 2005). In this view, it appears urgent to find biological markers which can go beyond classical behavioral assessment to detect even minor cognitive alterations. These new tools would help clinicians to identify which patients are more at-risk to develop or extend psychopathologies, and would thus significantly improve treatment through best suited medication as well as specialized and individualized cognitive rehabilitation programs (Campanella, 2016).

In this topic, our aim is to illustrate how and why cognitive event-related potentials (ERPs) may help, across various psychopathological populations, to specify the neuro-cognitive alterations presented by each patient in order to adapt the treatment. With this in mind, different authors will describe how ERPs may be helpful to better understand the pathophysiological mechanisms involved in diverse mental diseases and to adapt the therapeutic proposals accordingly. In this view, discriminating early and late ERPs modifications is thought to be of the greatest relevance in child psychopathology (Chronaki). A major focus on ERP correlates of attentional control is presented as a crucial aspect in child social anxiety (Wauthia and Rossignol). It is also suggested to include self-referential negative contexts when studying ERP correlates of adult social anxiety (Wieser and Moscovitch), while Cao et al. proposed a major focus on social feedback processes. Combining cognitive training and neuromodulation is also thought to have a positive impact on ruminations in major depression by increasing ERPs subtending inhibitory processes (Monnart et al.). Electroencephalogram (EEG) is also presented as a useful tool to predict outcome treatment in obsessive-compulsive disorders (Krause et al.). Moreover, Brion et al. suggested that ERPs may help to differentiate the successive steps, envisaged as a continuum, leading patients from an addictive state (alcohol-dependence) to Korsakoff syndrome. Finally, combining ERPs with hemodynamic data may help to better tag to pathological emotional disturbances indexing schizophrenia (Balconi et al.).

Overall, the rationale of this approach is that a better understanding of the underlying brain neurophysiological activities, by means of ERPs which are a quite cheap and easy to implement tool, could be highly useful to clinicians to install a best suited individualized treatment (specifically addressing the individual deficits of the patient). Two other papers finally propose to go one step further by presenting new “perspective” tools offering innovative possibilities to further extend the understanding of the electrophysiological correlates of psychopathological states. First, Karch et al. shed light on the measurement of neural oscillations to enlight the understanding of intentional actions. Second Schröder et al. suggest that the “bimodal” P300 component could offer an interesting add-on tool in a near future to enhance our understanding of the pathophysiology subtending mental diseases. We thus hope that this research topic will simultaneously illustrate: (1) *what can already be done*, i.e., the direct potential outcomes that can be expected right now from developing the use of electrophysiology in clinical psychopathology; (2) *what will soon be possible*, particularly following the development of new methodological and experimental proposals which will offer new perspectives for clinicians.

## Author contributions

All authors listed have made substantial, direct and intellectual contribution to the work, and approved it for publication.

### Conflict of interest statement

The authors declare that the research was conducted in the absence of any commercial or financial relationships that could be construed as a potential conflict of interest.
